# Opportunistic Screening for Bone Demineralization by Whole Body CT in a Red‐Handed Howler Monkey (*Alouatta belzebul*) Under Human Care

**DOI:** 10.1111/jmp.70107

**Published:** 2026-08-21

**Authors:** Thaiza H. T. Fernandes, Ieverton C. C. Silva, João Marcelo Azevedo de Paula Antunes, Rhaysa Allayde Silva Oliveira, Julianne Moura da Silva, Yumma Bernardo Maranhão Valle, Webert Aurino Silva, Fabiano Séllos Costa

**Affiliations:** ^1^ Radiology Sector Focus Diagnóstico Veterinário Recife Brazil; ^2^ Veterinary Hospital Federal Rural University of Semi‐Arid Mossoró Brazil; ^3^ Wildlife Medicine Division Dois Irmãos Zoo Recife Brazil; ^4^ Department of Veterinary Medicine Federal Rural University of Pernambuco Recife Brazil

**Keywords:** captive primates, imaging diagnostic, metabolic bone disease, QCT, tomography, trabecular bone

## Abstract

An eight‐year‐old male red‐handed howler monkey ( *Alouatta belzebul* ) underwent whole‐body computed tomography for gastrointestinal signs. Quantitative CT of the lumbar vertebrae opportunistically revealed severe bone hypoattenuation, demineralization, and deteriorated trabecular architecture. This incidental diagnosis of metabolic bone disease was directly attributed to chronic inadequate management conditions, specifically a severely imbalanced diet and insufficient sunlight exposure in captivity.

**Abbreviations**
BMD: bone mineral density
CT: computed tomography
HU: Hounsfield units
QCT: quantitative computed tomography
ROI: region of interest
vBMD: volumetric bone mineral density

## 1 Case Report

An eight‐year‐old, intact male red‐handed howler monkey ( *Alouatta belzebul* ) from the Dois Irmãos State Park Zoo in Recife, Brazil (8°04′03″ S, 34°55′00″ W), presented with a two‐day history of foul‐smelling watery diarrhea, lethargy, and appetite loss. Complete blood count and serum biochemical analyzes (urea, creatinine, ALT, and AST) were within normal reference ranges [1]. Parasitological fecal examination was negative.

**FIGURE 1:**
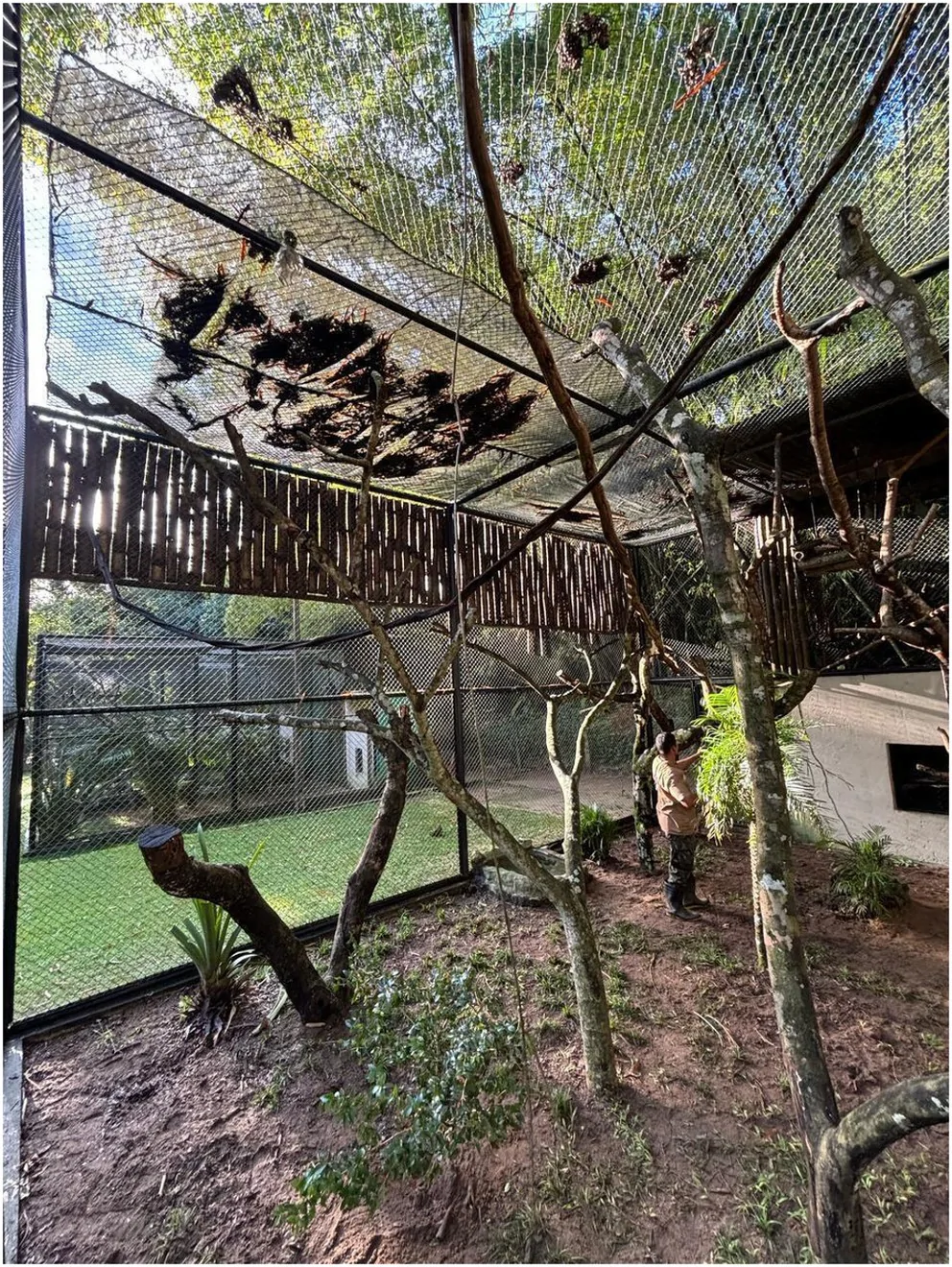
Photographic view of the enclosure where the red‐handed howler monkey ( *Alouatta belzebul* ) was maintained. The environment features significant shaded areas, limiting the animal's exposure to natural unfiltered sunlight necessary for cutaneous vitamin D3 synthesis.

The monkey had been rescued as an infant and maintained at the zoo since early life. The enclosure comprised an area of 10 m^2^ and a height of 4 m, with limited sunlight exposure and a lack of artificial lighting (Figure 1). The diet consisted of commercial chow for medium‐sized primates, bananas, green leaves offered three times a day, and additional fruits provided in the early afternoon. Due to the retrospective nature of this case, the precise daily intake quantities in grams (g/day) were not available in the historical records. Despite the provision of commercial feed and leaves, dietary imbalances coupled with environmental limitations likely compromised the animal's calcium metabolism. For further investigation, the animal was referred to a veterinary imaging center for electrocardiography, abdominal ultrasonography, and non‐contrast whole‐body computed tomography (CT). Due to the retrospective nature of this study, animal research ethics committee approval was not required; clinical data and anonymized images complied with institutional policies.

**FIGURE 2:**
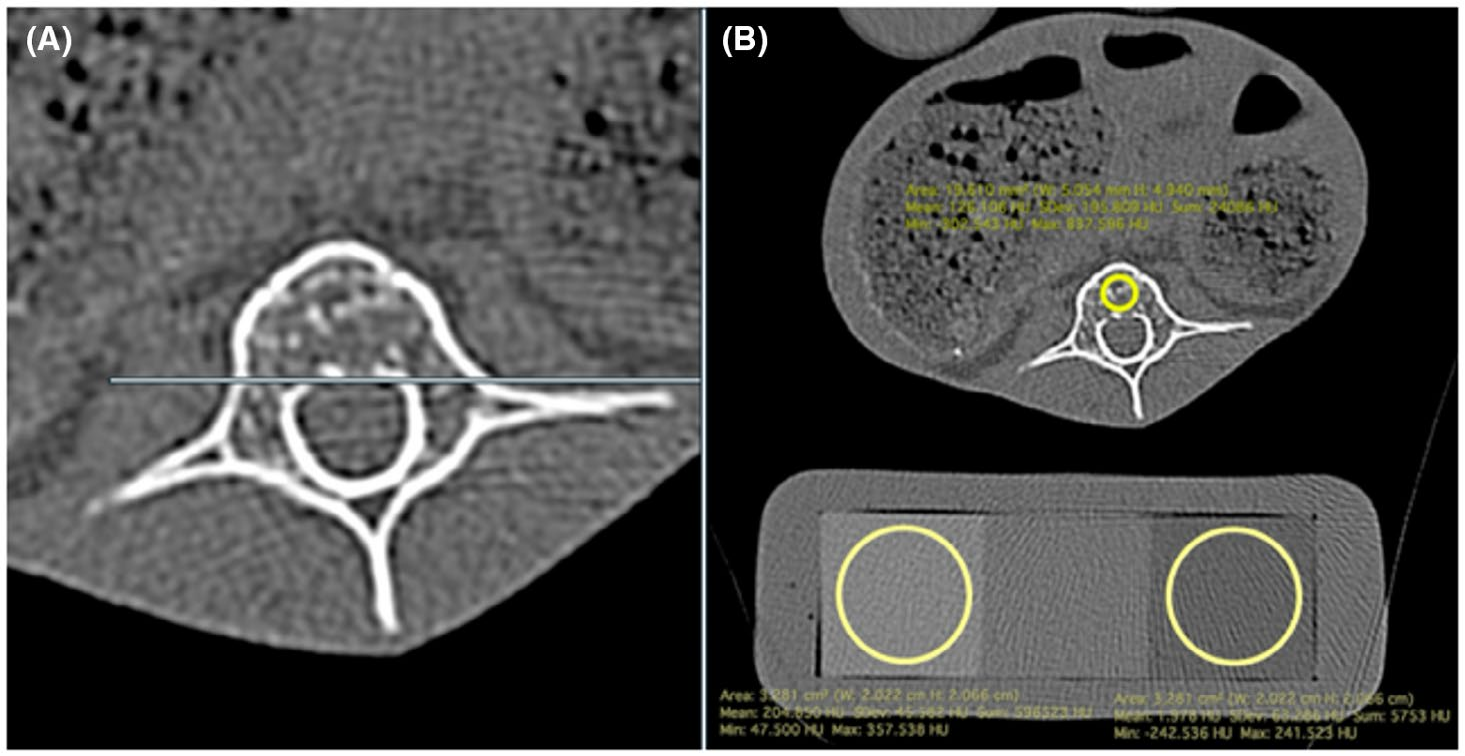
Cross‐sectional tomographic images of the second lumbar vertebra of a red‐handed howler. Bone filter. (A) Hypoattenuation and degradation of trabecular bone architecture. (B) ROI selection in the trabecular bone of the vertebra (small circle) and ROI selection in the QCT Phantom (large circles).

Procedures were performed under general anesthesia induced with intramuscular ketamine (10 mg/kg) and midazolam (1 mg/kg) and maintained with isoflurane via face mask. Non‐contrast helical CT was performed using a Bright‐Speed scanner (General Electric, Fairfield, CT, USA) with 2‐mm slice thickness, 1‐mm reconstruction interval, 120 kV, and automatic tube current modulation. Bone and soft tissue algorithms were applied (WW: 1400, WL: 300). The monkey was positioned in ventral recumbency, and a quantitative CT (QCT) phantom (IA Image Analysis Inc.) was placed ventrally at the lumbar level and scanned simultaneously (Figure 2). Images were reviewed using Horos software (version 3.3.5).

**FIGURE 3:**
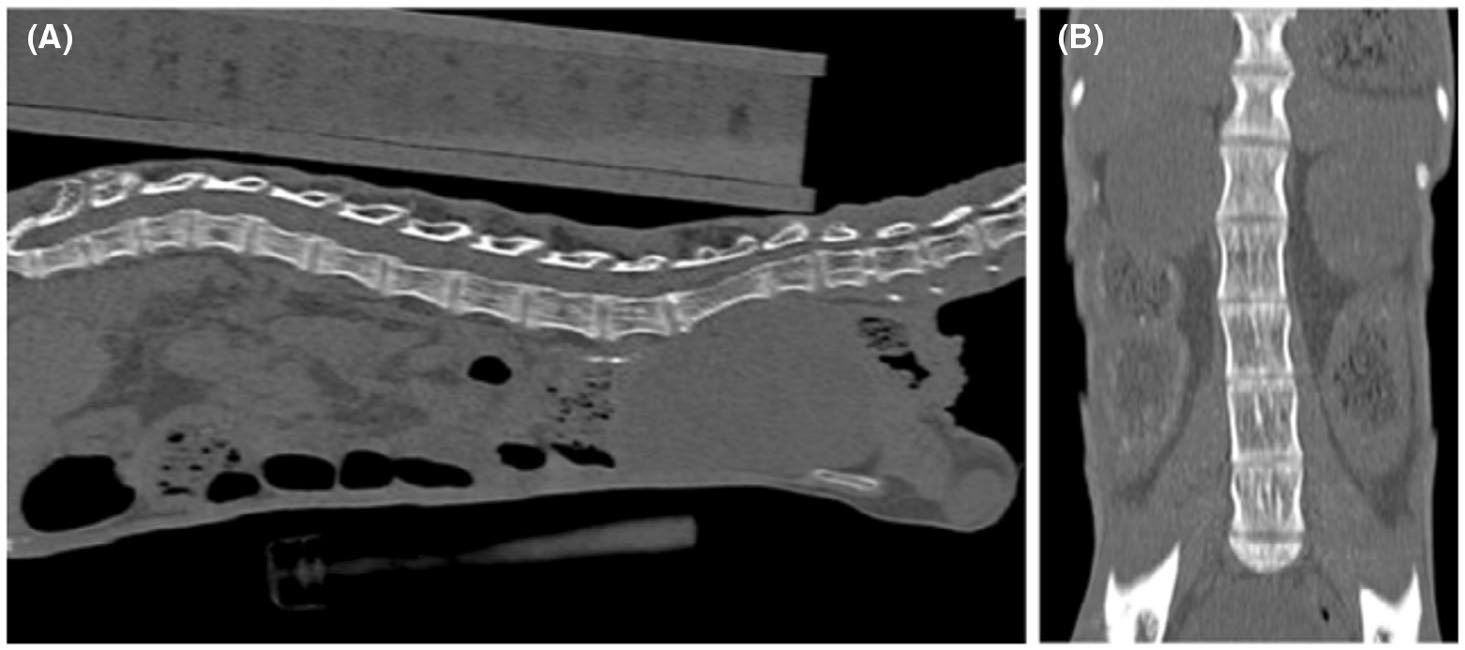
Multiplanar reconstruction of the lumbar spine in a red‐handed howler. Bone filter. Sagittal (A) and dorsal (B) views showing the lumbar spine and vertebral trabecular architecture degraded (arrows).

The primary clinical indication for CT was suspected hepatic lipidosis, but liver attenuation was normal (56–60 HU). Given the limited sunlight exposure, the CT provided an opportunity to evaluate bone tissue. Circular regions of interest (ROIs, ~20 mm^2^) were placed within the trabecular bone of the first through fifth lumbar vertebral bodies to obtain radiodensity measurements in Hounsfield units (HU) using multiplanar reconstructions for standardization (Figure 3).

Radiodensity values were recorded for trabecular bone (HUt), and for the water (HUw) and bone (HUb) sections of the QCT phantom (Table 1). Bone mineral density (BMD), expressed in mg/cm^3^, was calculated using the equation: BMD = 200 HUt / (HUb—HUw), as previously described [2, 3, 4, 5].

**TABLE 1:** Radiodensity and bone mineral density obtained by computed tomography scans of the trabecular bone of lumbar vertebrae in a red‐handed howler with metabolic bone disease.

Lumbar vertebrae	Radiodensity (HU)	BMD (mg/cm^3^)
L1	194.74	193.89
L2	170.39	168.42
L3	140.95	138.86
L4	129.69	128.41
L5	133.86	132.34
Mean ± SD	153.93 ± 24.88	152.38 ± 25.06

Abbreviations: BMD, bone mineral density; HU, Hounsfield units; SD, standard deviation.

Subjective evaluation revealed severe hypoattenuation, demineralization, and deterioration of the trabecular architecture across all lumbar vertebrae, consistent with metabolic bone disease. No pathological fractures were identified.

## 2 Discussion

The markedly low lumbar trabecular BMD values observed support a diagnosis of severe bone demineralization compared to other primate benchmarks [6], highlighting a pathology that appears to be rarely documented in *Alouatta belzebul* . To better illustrate the severity of the bone loss in this case, Table 2 compares the lumbar trabecular BMD values obtained from our patient with established reference values for healthy adult *Macaca fascicularis* (Cynomolgus monkeys) [6]. The substantially lower values found in the present case support the diagnosis of severe bone demineralization. Given that this endemic species is classified as vulnerable due to habitat fragmentation [7], identifying captivity‐induced metabolic disorders is essential to enhance diagnostic standards and optimize the clinical management of zoo‐housed populations.

**TABLE 2:** Comparison of lumbar trabecular bone mineral density (BMD) between the *Alouatta belzebul* patient and reference values for healthy *Macaca fascicularis* .

Subject	Species	Mean lumbar trabecular BMD (mg/cm^3^)	References
Present case (8 years)	*Alouatta belzebul*	152.38 ± 25.06	Current study
Healthy young (8–10 years)	*Macaca fascicularis*	433.08 ± 51.44	Zeng et al. (2023) [6]
Healthy older (≥ 20 years)	*Macaca fascicularis*	337.14 ± 61.94	Zeng et al. (2023) [6]

Maintaining primates in zoos and conservation centers poses challenges due to difficulties in replicating their natural habitat and preventing chronic stress, which can contribute to a variety of health problems, including metabolic bone disease, previously reported in several non‐human primate species [8, 9]. This condition may result in osteopenia/osteoporosis, osteomalacia, and rickets [9]. In the present case, bone demineralization was likely influenced by nutritional deficiencies and inadequate husbandry practices. Limited dietary vitamin D intake and insufficient exposure to sunlight appear to have been key factors. New World primates are particularly susceptible to metabolic bone disease because they cannot utilize vitamin D_2_ and instead rely on the cutaneous conversion of provitamin D_3_ to previtamin D_3_ for vitamin D synthesis and calcium regulation [9, 10].

Quantitative computed tomography (QCT) has been shown to have higher sensitivity than dual‐energy X‐ray absorptiometry (DXA) for diagnosing osteoporosis [11], as it measures true volumetric bone mineral density (vBMD), which is size‐independent and unaffected by calcifications, osteophytes, or spinal deformities. Non‐human primates have been widely used as experimental models for human osteoporosis research, and QCT has been applied in many such studies, although most have involved Cynomolgus monkeys. No previous densitometric studies have been reported for howler monkeys [6]. In Cynomolgus monkeys aged 8–12 years, mean trabecular lumbar BMD has been reported as 428.05 ± 53.75 mg/cm^3^ [6]. The substantially lower values found in the present case support the diagnosis of bone demineralization (Table 1).

For osteodensitometric evaluation, the lumbar vertebrae were selected, as is standard in both human patients and experimental primate studies [12, 13, 14, 15, 16]. Although anatomical data for *A.belzebul* are scarce, a study on four adult male cadavers described the species as having seven cervical, thirteen thoracic, five lumbar, and three fused sacral vertebrae [17]. Trabecular bone is often the focus of BMD studies because its remodeling rate is four to eight times higher than that of cortical bone, owing to its larger surface area in contact with hormones and drugs, making it more sensitive to bone mass loss [2, 4, 18]. Compared to other densitometric techniques, QCT allows separate assessment of trabecular bone, a distinction not possible with DXA [19].

In this study, a QCT imaging phantom was used to correct trabecular bone attenuation values in Hounsfield units (HU). HU measurements for a given material can vary significantly between CT scanners; depending on beam energy (kVp) and material composition, differences of 7–56 HU have been reported. Multiple factors such as kVp, mA, reconstruction algorithm, and slice thickness can also influence attenuation values, even within the same scanning protocol, highlighting the need for standardized adjustments [11, 12, 13, 14, 16, 20].

Opportunistic screening via QCT leverages imaging data acquired for unrelated clinical indications to evaluate BMD [20]. This method enables earlier detection and intervention without additional imaging, radiation exposure, or appointments, potentially preventing fractures [11, 12, 13, 14]. Here, an abdominal CT scan intended to evaluate the liver led to the incidental diagnosis of metabolic bone disease. Following such imaging findings, complementary evaluation of serum calcium, phosphorus, electrolytes, 25‐hydroxyvitamin D, and parathyroid hormone (PTH) is crucial to investigate nutritional secondary hyperparathyroidism, a common consequence of inadequate UVB exposure and dietary imbalances in captive New World primates [21]. To halt disease progression, immediate interventions are required, including correcting the dietary calcium‐to‐phosphorus ratio, initiating oral vitamin D3 and calcium supplementation, and ensuring regular access to unfiltered sunlight or full‐spectrum artificial UVB lighting [9, 10]. This highlights the value of opportunistic assessments in wildlife medicine for early intervention, optimization of captive management, and enhancement of animal welfare under human care.

## Conflicts of Interest

The authors declare no conflicts of interest.

## Data Availability

The data that support the findings of this study are available from the corresponding author upon reasonable request.
